# Nail-wounds of the Feet of Horses

**Published:** 1898-10

**Authors:** Roscoe R. Bell

**Affiliations:** Brooklyn, N. Y.


THE JOURNAL
OF
COMPARATIVE MEDICINE AND
VETERINARY ARCHIVES.

VOL. XIX. *OCTOBER, 1898.* No. 10.

### NAIL-WOUNDS OF THE FEET OF HORSES.

BY ROSCOE R. BELL, D.V S.,
BROOKLYN, N. Y.

I READ a short paper at the Omaha meeting of the United States Veterinary Medical Association upon the subject of "Acute Indigestion in Horses," and I selected that disease because I considered it most frequently met with in private practice of any we are called upon to prescribe for.

It strikes me that we should know as much about our common diseases as is possible,and should not always be discussing speculative and rare conditions which are interesting chiefly on account of our unfamiliarity with them. That reminds me of a dealer in horse-feed who recently became a successful bidder for supplying one of the departments of Brooklyn. The official who had the disposal of the contract was innocent of any practical knowledge of the subject, and was struck by the liberality of the bid, while the initiated ones were laughing at the imposition. The specifications called for bids for furnishing oats, hay, bran, and oil-meal. When the bids were opened the majority of the competitors gave a legitimate price for each article called for; but when the bid of the individual whose credulity is under discussion was inspected it was found that his figures on oil-meal and bran were ridiculously low, but when it came to oats and hay he was considerably higher than any of his competitors. To the official his bid was the winner; but the other bidders would gladly have presented the department with what little bran and meal they might require during the year, since the bulk of the requisition to be consumed would necessarily be oats and hay. So, too, it seems to me that we are often straining to solve uncommon diseases and conditions

[1] Read before the New York State Veterinary Medical Society, New York City, N. Y., September 15, 1898.

to the neglect of those from which we derive our daily bread and through which our local reputations are made or unmade.

For this reason I bring before you the subject of punctured wounds of the feet of horses, with special reference to their successful treatment according to known methods of antiseptic surgery and therapeutics. For the purpose of saving time in the reading of this paper, and leaving plenty of room for discussion by our members, I shall omit any extended review of the anatomy of the foot, further than to mention those structures most likely to be injured or invôlved in the inflammation consequent upon the traumatism. Viewing the plantar aspect of the horse's foot, we observe the sole occupying the majority of the surface, somewhat heart-shaped, the triangle at its base being filled by the horny frog, and its sides and apex surrounded by and united with the wall, which is duplicated along the side of the frog to form the bars. Beneath the sole at all points is the laminæ or velvety tissue, which is interposed between the horn of the sole and the inferior face of the os pedis. Beneath the horny frog there is the fibrous or fatty cushion, a thick elastic body intended by nature to protect the delicate structure of the navicular apparatus and the inferior attachment of the perforans tendon. The distance a nail would have to penetrate the sole before it would injure any of these structures would depend upon its point of entrance. Thus, if anterior to the point of the frog the distance to the sole of the os pedis is not more than the half an inch in most feet, while at the posterior third of the frog two inches is about the distance necessary to traverse before reaching the navicular joint. The most prolific causes of these wounds are the stepping upon nails in the streets and from pulling the shoe half off, which shifts its position, and the nails are again driven into the sole. The former are termed "gathered nails," the latter "drawn nails." Drawn nails mostly enter the sole near the wall, and are not so serious as gathered nails. They seldom pass through the lamina, and when they do the inferior face of the os pedis receives the force of the nail-point, which, being necessarily blunt, a bruise to the periosteum, with localized periostitis, is the usual consequence, necessitating a longer time to recover, but which is almost certain to follow properly directed curative measures. The most selective point for gathered nails is, in my judgment, upon the sides of the frog near the blending with the bars, at a point one inch posterior to its apex, the direction upward and backward. If the penetration is very deep and the point directed backward the navicular joint may be reached; if the point be directed more perpendicularly, the semilunar crest of the os pedis receives the injury, and in the inflammation which ensues the terminal fibres of the perforans tendons are involved, which may be determined largely by the absence of the lancinating pain that would accompany the joint involvement, and confirmed by the persistency with which the animal walks upon the toe of the foot, in some cases pivoting on the very apex. If the penetration is not sufficiently deep to have reached these structures, it has expended itself in the fatty frog, and the prognosis is more favorable and the period of indisposition less prolonged. Very many punctured wounds do not cause any degree of lameness after withdrawing of the offending body, even though the lamina has been injured, as evidenced by the copious hemorrhage, and, as a matter of clinical experience, a lesser degree of lameness may be anticipated when the hemorrhage is profuse than when it does not occur. This assertion may be explained upon the hypothesis that in one instance the blood which escaped from the injured vessels ran out of the wound, while in the other it is confined inside of the wall and undergoes degeneration due to microbic infection. The old theory that punctured wounds causing lockjaw were due to injury of the nerves must now be relinquished in the light of bacteriological discovery, which holds that the bacillus of tetanus usually comes from the earth, enters the system through the abrasion of the sole, and sets up a ptomaïn-producing process. I will not contend that the disease does not develop more rapidly when the nerve-sheaths are injured, but they will develop in other tissues. I have been enabled, however, to state to a client that his horse was in no danger of lockjaw from the wound in the foot if it was freely suppurating. Did any gentleman present ever see a case of tetanus follow a nail-wound of the foot where free suppuration was present or had existed? Is not the history invariably that the wound was insignificant and no pus was present?

The therapeutics of punctured wounds of the feet has undergone a radical change in the past decade. For an attendant to have omitted to employ a hot oil-meal poultice to such a condition, no matter what the state of the pathological process, would have been to exhibit extreme ignorance of the fundamental law; but I am glad to say that veterinarians are now using their knowledge and reason, omitting hard-and-fast rules and adopting such measures as are demanded by the conditions present. Antisepsis has taken the place of ignorance, with the happiest results. A veterinarian called to see a patient from whose foot has just been withdrawn a gathered or drawn nail will have the foot thoroughly washed and dried; pare down the sole or frog, as the case may be, enlarge the opening sufficiently to admit a clean probe, by which he will be enabled to discover to what depth the puncture has been made, and compare the distance on his probe to the visible signs upon the nail (being careful to observe that no portion of the nail has been left in the wound). The wound will then be thoroughly sterilized and neatly dressed in the manner which he has adopted as serving the purpose best in his judgment. My own method is by the use of oakum. A half-pail solution is made of some good antiseptic bichloride, creolin, carbolic acid, sanitas, or whatever has given the best results in his hands. Into this is placed enough oakum to nicely envelope the foot (sole, wall, and heel), and thoroughly saturated, after which the foot is encased in it, and it is held in place by a piece of burlap cut in proper shape, and neatly bandaged by three three-yard, two-inch domet-cloth bandages, making a very surgical-appearing dressing which retains its position as long as circumstances require it. Now, the attendant is ordered to prepare a similar solution every three, four, six or ten hours, as you may wish, and place the dressed foot in it and allow it to remain for a few minutes. In many instances it is inconvenient to have dressings removed, and this method effectually does away with the necessity. If warm poultices are indicated, the solution may be made with hot water, and the dipping of the encased foot is equivalent to a hot antiseptic poultice; if cold applications are the indication, the substitution of cold water, medicated by the addition of an approved antiseptic, will be a submersion of the most approved method. When the surgeon removes the dressing the wound is perfectly clean and aseptic.

Whatever good results are to be obtained from the aseptic conception of treating traumatisms may be secured by these means. Complications and unusual conditions are to be met by the laws of common sense, based upon a knowledge of pathology. The frequent involvement of the terminal fibres of the perforans tendon is best treated by the application of the high-heeled shoe, to throw the parts at rest. When the wound has healed, the animal still walking upon his toe, the high-heeled shoe should remain on the foot, and the coronet blistered; if an improvement takes place in a slight degree, lower the heel some and reblister, and, if necessary, turn the horse to pasture, still wearing the elevated heel, in full confidence that recovery will eventually occur. In cases of joint involvement, continued cold irrigation by antiseptic solution will yield the best results, and when the acute symptoms have subsided blistering of the coronet will send the case further on toward recovery. In case of puncture of the sole of the os pedis, and protracted lameness, it may be necessary to pare down, remove the laminæ, and scrape the bone, always adhering to the principle of antisepsis.

